# Comparison of primary coronary percutaneous coronary intervention between Diabetic Men and Women with acute myocardial infarction

**DOI:** 10.12669/pjms.312.6646

**Published:** 2015

**Authors:** Heng-Liang Liu, Yang Liu, Zhen-Xuan Hao, Guo-Ying Geng, Zhi-Fang Zhang, Song-Bin Jing, Ning Ba, Wei Guo

**Affiliations:** 1 Heng-Liang Liu, Department of Cardiology, The People’s Hospital of Zhengzhou, Zhengzhou 450002, Henan, China; 2 Yang Liu, Department of Cardiology, The People’s Hospital of Zhengzhou, Zhengzhou 450002, Henan, China; 3 Zhen-Xuan Hao, Department of Cardiology, The People’s Hospital of Zhengzhou, Zhengzhou 450002, Henan, China; 4 Guo-Ying Geng, Department of Cardiology, The People’s Hospital of Zhengzhou, Zhengzhou 450002, Henan, China; 5 Zhi-Fang Zhang, Department of Cardiology, The People’s Hospital of Zhengzhou, Zhengzhou 450002, Henan, China; 6 Song-Bin Jing, Department of Cardiology, The People’s Hospital of Zhengzhou, Zhengzhou 450002, Henan, China; 7 Ning Ba, Department of Cardiology, The People’s Hospital of Zhengzhou, Zhengzhou 450002, Henan, China; 8 Wei Guo, Southern Medical University, The People’s Hospital of Zhengzhou, Zhengzhou 450002, Henan, China

**Keywords:** Acute myocardial infarction, Diabetes mellitus, primary percutaneous coronary intervention, Female

## Abstract

**Objective:**

This study aimed to explore the short-term efficacy and safety of primary percutaneous coronary intervention (PCI) in female diabetic patients complicated with acute myocardial infarction (AMI).

**Methods:**

A total of 169 diabetic patients with AMI who underwent primary PCI were selected and divided into group A (52 females) and group B (117 males). The clinical data, characteristics of coronary artery lesions, lengths of hospital stay, and incidences of complications were then compared between two groups.

**Results:**

The average age, history of hyperlipidemia, double branch lesions, triple branch lesions, and left main lesions were significantly higher in group A than in group B (P < 0.05). Smoking history, PCI history, and pre-infarction angina were distinctly lower in group A than in group B (P < 0.05). Thrombolysis in myocardial infarction 3 (TIMI3) flow and TIMI myocardial perfusion grade 3 (TMPG3) after PCI were markedly lower in group A than in group B (P < 0.001). Group A had a higher incidence of complications, such as severe arrhythmia, cardiac function Killip III/IV, cardiogenic shock, major, moderate and mild bleed event, as well as a 30-day mortality rate, compared with group B (P < 0.05).

**Conclusion:**

In summary, our study demonstrated that female diabetic patients with AMI had lower TIMI3 flow and TMPG3 following PCI than male patients, while there was higher incidence of complications and 30-day mortality rate. Therefore, more attention should be paid to the therapy of diabetic women with acute myocardial infarction as well as the control of risk factors.

## INTRODUCTION

Primary percutaneous coronary intervention (PCI) is the primary procedure used to rescue patients with acute myocardial infarction (AMI).^1^ Abnormal glucose metabolism is one of the major risk factors of the initiation and development of arteriosclerosis,^2^,^3^ and diabetes is considered a risk equivalent of coronary heart disease (CHD). The incidence of major adverse cardiac events (MACE) in patients with diabetes mellitus (DM) complicated with AMI treated by primary PCI is significantly higher than in non-diabetic patients.^3^-^5^ Studies have shown that the curative effect of primary PCI is better than that of thrombolytic therapy in women with diabetes complicated with AMI.^2^ However, female AMI patients experience special physiological and pathological changes different from males,^2^ particularly females complicated with diabetes. Therefore, the efficacy and complications of primary PCI need to be investigated. Accordingly this study aimed to analyze the efficacy and safety of primary PCI in different-gender patients with diabetes complicated with AMI.

## METHODS

### Subjects

A total of 169 diabetic patients complicated with AMI who underwent primary PCI in the coronary care unit between Jan 2010 and June 2014. These patients were divided into group A (observation group; 52 females aged 55-81 years, average age = 69.5 ± 9.8 years) and group B (control group; 117 males aged 42-75 years, average age = 69.5 ± 6.8 years). The diagnostic criterion of AMI was based on the ACC/AHA 2007 guidelines for the Management of Patients with ST-Elevation Myocardial Infarction.^6^ Diagnostic criteria for DM were based on the American Diabetes Association diabetes diagnostic criteria.^7^ The inclusion criteria were 1) onset of STEMI within 12 h, and 2) patients agreed to primary PCI. The exclusion criteria were 1) onset of STEMI >12 h, 2) patients were suspected with aortic dissection, 3) remedial PCI after thrombolysis treatment, and 4) severe hepatorenal dysfunction. This study was conducted in accordance with the declaration of Helsinki with approval from the Ethics Committee of the People’s Hospital of Zhengzhou. Written informed consent was obtained from all participants.

### Primary PCI

After hospital admission, the patients were subjected to 18-lead electrocardiography (ECG) followed by electrocardiograph monitoring; oxygen inhalation, blood glucose, blood lipid, myocardial enzyme, and troponin tests; and other related biochemical and routine tests. At the same time, the patients took 300 mg of aspirin and 600 mg of clopidogrel by chewing. The Judkins Technique of coronary angiography was used, in which the catheter was guided through the blood vessel to the opening of coronary arteries. The patients received 3000 and 7000 U heparin through a sheathing canal before and after angiography. A thrombus suction catheter was used to remove the thrombus if a thrombus shadow was visible after angiography, and then a guide wire was passed through the culprit vascular lesions. When necessary, tirofiban was injected into coronary arteries with a dose of 10µg/kg within 3min, followed by continuous intravenous (IV) administration at 0.15µg/(kg•min) by pumping for 24h. Coronary angiography was repeated to check coronary blood flow. Percutaneous transluminal coronary angioplasty with stent implantation or primary stenting was performed depending on the disease condition. The levels of myocardial enzymes and troponin, ECG, echocardiography, as well as hepatorenal function were reviewed after procedure. The patients continued to take 100 mg/day aspirin, 75 mg/day clopidogrel, statins, β-receptor blockers, and hypoglycemic agents. Only culprit vessels were treated during primary PCI, and other lesion vessels (if any) were treated by a second procedure 7-14 days after PCI.

### Observation indices

Coronary arteriography was performed to determine the characteristics of lesions in the two groups, including the length of hospital stay, average time from admission to balloon dilatation, number of patients implanted with more than two stents during primary PCI, second PCI during hospitalization, angina after infarction, re-infarction, acute and subacute thrombosis stent, severe arrhythmia, cardiac function Killip class III/IV and cardiogenic shock, 30-day mortality rate, Major bleed event (intracranial hemorrhage, gastrointestinal tract hemorrhage, massive hemoptysis caused by hemodynamic instability, decrease in hemoglobin ≥5 g/dL, decrease in hematocrit ≥15%); moderate bleeding (hemoptysis, hematemesis ≥100 mL/day, melena, and gross hematuria); and mild bleeding (hemoptysis, hematemesis <100 mL/day, hematoma after puncture, skin ecchymosis, mucosal and gingival bleeding, and microscopic hematuria).etc. Thrombolysis in myocardial infarction (TIMI) trial and TIMI myocardial perfusion grade (TMPG) of infarction-related blood vessels after PCI were recorded.^8^,^9^

### Statistical analysis

The statistical software SPSS16.0 was used to analyze all data, which were presented as *x* ± *s*. Enumeration and measurement data were analyzed by the *χ*^2^ test and *t* test, respectively. *P* < 0.05 was considered as a significant difference.

## RESULTS

### General information

No significant differences were observed in the history of hypertension, serum creatinine, and history of CHD between the two groups (*P* > 0.05). The age and history of hyperlipidemia were higher in group A than in group B (*P* < 0.001, *P* < 0.01), whereas the smoking history, PCI history, and pre-infarction angina were remarkably lower in group A than in group B (*P* < 0.01, Table-I).

**Table-I:** Comparison of general data between two groups.

Item	Female group (52 cases, group A)	Male group (117 cases, group B)	P value
Age (years old, x±s)	69.5±9.8	56..3±9.6	0.001
Hypertension [cases (%)]	37(71.15)	87(74.36)	0.664
Smoking history [cases (%)]	7(13.46)	86(73.5)	0.000
Hyperlipemia [cases (%)]	36(69.23)	53(45.30)	0.004
Serum creatinine (mmol/L)	90.7±12.6	88.9±15.3	0.837
PCI history [cases (%)]	4(7.69)	26(22.22)	0.023
Preinfarction angina [cases (%)]	3(5.77)	23(19.66)	0.021
History of CHD [cases (%)]	4(7.69)	11(9.40)	0.718

### Coronary artery lesions

Single branch lesions were significantly lower (*P* < 0.01) but double branch lesions, triple branch lesions, and left main lesions were distinctly higher (*P* < 0.05) in group A than in group B. No statistical differences were observed in the target vessels treated by primary PCI and TIMI0/1 before PCI, GPIIb/IIIa inhibitor used between the two groups (*P* > 0.05). TIMI3 flow after PCI was markedly lower in group A than in group B (*P* < 0.001). TMPG0/1 and 2 were higher (*P* < 0.05) and TMPG3 was evidently lower (*P* < 0.001) in group A than in group B (Table-II).

**Table-II:** Comparison of characteristics of coronary artery lesions between two groups [cases (%)].

Characteristics of lesions	Female group (52 cases, group A)	Male group (117 cases, group B)	P value
Access site(right radial artery)	49(94.23)	112(95.73)	0.976
GPIIb/IIIa inhibitor use	32(61.54)	54(46.15)	0.065
Single branch lesions	5(9.62)	58(49.57)	0.000
Double branch lesions	23(44.23)	32(27.35)	0.031
Triple branch lesions	24(46.15)	27(23.08)	0.002
Left main lesions	8(15.38)	6(5.13)	0.025
Target vessels in primary PCI			
LAD	27(51.92)	65(55.56)	0.661
LCX	11(21.15)	25(21.37)	0.975
RCA	14(26.92)	27(23.07)	0.590
TIMI grade			
Preoperative TIMI 0~1	50(96.15)	112(95.73.)	0.897
Postoperative TIMI3	38(73.08)	109(93.16)	0.000
Postoperative TMPG grade			
grade 0~1	10(19.23)	8(6.84)	0.015
grade 2	10(19.23)	10(8.55)	0.047
grade 3	32(61.54)	99(84.62)	0.000

### Balloon dilatation

No statistical differences were observed in the average time from admission to hospital to balloon dilatation, number of patients with two stents, second PCI, and incidence of stent thrombosis (*P* > 0.05). Significant decrease were detected in the rates of post-infarction angina, average length of hospital stay, re-infarction, severe arrhythmia, cardiac function Killip III/IV cardiogenic shock, major, moderate and mild bleed event, meanwhile there was a more notable decrease of 30-day mortality in group B (*P* < 0.05, Table-III).

**Table-III:** Comparison of the length of hospital stay, PCI characteristics and incidence of complications between two groups.

Item	Female group (52 cases, group A)	Male group (117 cases, group B)	P value
Average length of hospital stay (d)	15.2±3.6	10.1±2.7	0.037
Average time from admission to hospital to balloon dilatation (min)	93.2±23.8	95.6±17.6	0.695
Number of patients with two stents [cases (%)]	36(69.23)	73(62.39)	0.391
Second PCI [cases (%)]	38(73.07)	75(64.10)	0.252
Post-infarction angina [cases (%)]	17(32.69)	22(18.80)	0.047
Re-infarction [cases (%)]	8(15.38)	6(5.13)	0.025
Stent thrombosis [cases (%)]	3(5.77)	1(0.85)	0.164
Severe arrhythmia [cases (%)]	19(36.54)	9(7.69)	0.000
Major bleed event [cases (%)]	5(9.61)	2(1.71)	0.017
Moderate and mild bleed event [cases (%)]	7(13.46)	5(2.94)	0.032
Cardiac function Killip III~IV [cases (%)]	12(23.08)	8(6.84)	0.002
Cardiogenic shock after PCI [cases (%)]	5(9.61)	2(1.71)	0.046
30-day mortality rate [cases (%)]	5(9.62)	1(0.85)	0.016

## DISCUSSION

CHD is the primary cause of death in women,^8^,^9^ whose pathophysiology, clinical manifestation, as well as short- and long-term treatment efficacies, differ from those of men.^3^ The most important therapeutic principle of AMI is the continuous and complete opening of infarction-related blood vessels as soon as possible.^1^ Primary PCI is superior to thrombolytic therapy in effectively reducing incidence of MACE for both female and male diabetic patients complicated with AMI.^10^,^11^

In the present study, the onset of AMI in females was 13 years later than that in males, whose blood lipid content was significantly lower than that of females. Elevated blood lipid level is considered as a major risk factor of the initiation and development of arteriosclerosis. Studies have shown that the late onset of CHD in female is due to the ability of estrogen to increase blood high-density lipoprotein cholesterol, which has a preventive effect on atherosclerosis. This mechanism gradually attenuates and even disappears after menopause. Therefore, the morbidity of CHD in female after menopause rapidly increases.^3^ The development of chest pains as an initial symptom is rare even in female patients with significant myocardial ischemia, particularly in female diabetic patients complicated with AMI, whose ailments are generally characterized by fatigue, shortness of breath, tiredness, and even heart failure, as well as myocardial infarction at diagnosis.^3^,^10^,^11^ In our study, pre-infarction angina was distinctly lower in females than in males, which may be correlated with atypical symptoms of angina pectoris in female patients. Given these unspecific and atypical symptoms, a small area of myocardial necrosis remains undetected in patients, which is also one of the reasons why females had lower PCI incidence than males in our study.

Euro Heart Survey has found that 37% patients with acute coronary syndrome (ACS) are already diagnosed with DM or diagnosed for the first time with DM.^12^ Moreover, diabetic patients experience more severe damage in the heart and vessels than non-diabetic patients, and their coronary artery disease always involves multiple blood vessels with diffuse lesions,^13^ which is usually complicated with microangiopathy and diabetic cardiomyopathy.^14^,^15^ Compared with non-diabetic patients, diabetic patients have higher incidence of re-infarction, heart failure, stroke, and death regardless of the phase (acute or chronic).^14^-^16^

In our research, the average length of hospital stay, post-infarction angina, re-infarction, severe arrhythmia and cardiac function Killip III/IV, cardiogenic shock, major, moderate and mild bleed event and 30-day mortality rate were dramatically higher in females than in males. The incidence of complications in female diabetic patients was higher than that in male patients because of the following reasons. First, women are attacked by pre-infarction angina less often than men, so they lack the protective effect of ischemic preconditioning, which results in a large infarction area. Second, females have atypical AMI symptoms and are usually diagnosed late, leading to relatively late opening of infarction-related arteries and a large infarction area.

Third, elevated blood sugar aggravates endothelial dysfunction, increases inflammatory reaction, exacerbates reperfusion injury induced by free radicals, and increases the excitability of β-receptors, which damage the myocardial cell membrane, upsets the balance of calcium inside and outside the membrane, and induces arrhythmia.^13^,^14^ Diabetic patients with AMI have relative or absolute lack of insulin and elevated plasma concentration of free fatty acids, which increase the oxygen consumption of damaged myocardium, expands the infarction area, aggravates ventricular remodeling, decreases cardiac function, induces heart failure and cardiogenic shock, as well as increases mortality.^15^,^16^

Fourth, microvascular dysfunction is more common in female diabetic patients with AMI. Blöndal et al.^3^ discovered that the mortality rate of female diabetic patients with AMI can reach 12%. TIMI3 and TMPG3 were lower in females than in males in our study. Restored forward flow of infarction-related arteries to TIMI3 is considered as the gold standard for successful reperfusion treatment. However, a significant difference exists in perfusion levels between peripheral coronary arteries because even the blood flow of an epicardial coronary artery achieves TIMI3. Research has demonstrated that myocardial tissues in 25%-30% of patients still experience insufficient reperfusion, i.e., no reflow and slow flow, when PCI restores epicardial blood flow to the normal level.^17^,^18^ In fact, successful reperfusion at the myocardial tissue level should be the final standard. TMPG, as a standard for perfusion at the myocardial level, includes the filling and emptying of contrast medium in the myocardium and can accurately evaluate the perfusion at the myocardial level. Diffuse lesions of epicardial coronary arteries in diabetic patients are always complicated with microangiopathy and microvascular dysfunction. In addition, hyperglycemia enhances inflammatory reaction and platelet-dependent microthrombosis, as well as weakens endothelium-dependent vasodilation, thereby aggravating perfusion disturbance of the coronary microcirculation.^5^ A number of studies have shown that the mortality of patients with TMPG0/1 is much higher than that of patients with TMPG2/3. Regarding patients with normal TIMI3 flow in epicardial coronary vessels, the incidence of slow flow and no reflow after primary PCI is markedly higher in female diabetic patients with AMI than in males.^19^

Fifth, diabetic females are more vulnerable to complications such as diabetic cardiomyopathy and poor cardiac function.^8^,^9^ Sixth, female diabetic patients complicated with AMI are older than males, so complications with diseases in other important organs that worsen bodily functions are common.^10^,^11^,^19^ Seventh, post-infarction angina and re-infarction are frequently observed in female diabetic patients with AMI. Considering that the coronary artery of females is relatively thinner than that of males, the incidence of in-stent restenosis is higher in females.^3^,^10^ D’Ascenzo et al.^10^ compared and analyzed 210 female and 623 male AMI patients after PCI procedure. They found that the morbidity of diabetes (female vs. male: 36.2% vs. 21.0%; *P* < 0.001) and hypertension (female vs. male: 82.3% vs. 73.7%; *P* = 0.006) are high and that no significant difference exists in the incidence of MACE between female and male patients. By contrast, the mortality rate of females is significantly higher than that of males^10^ (female vs. male: 20.0% vs. 8.1%; *P* = 0.029). Overall, more complications and higher mortality rate are observed in female diabetic patients with AMI after PCI surgery than in males.^19^

Fath-Ordoubadi et al.^11^ screened 1640 ACS patients who were implanted with a drug-eluting stent during PCI. They discovered that primary PCI is still the optimum choice for rescuing female diabetic patients with AMI even though risk factors such as diabetes or hypertension are more frequent in female patients than in younger males.

In conclusion, cardiologists should pay more attention to female patients as well as the prevention of female CHD and the control of risk factors because the symptoms of female AMI lack specificity. Moreover, the pathophysiology of female patients is unique. As soon as abnormal glucose metabolism is observed in female patients, active interventions through drugs and lifestyle changes are effective ways for reducing complications in female diabetic patients with AMI.
